# Development of Acceptable Quality Dose (AQD) and image quality-related diagnostic reference levels for common computed tomography investigations in a tertiary care public sector hospital of Khyber Pakhtunkhwa, Pakistan

**DOI:** 10.1007/s11604-024-01627-y

**Published:** 2024-07-27

**Authors:** Muhammad Yaseen, Tahira Nishtar, Mohammad Hassan Kharita, Banaras Khan, Shady AlKhazzam, Amir Ali, Laila Khan, Nasreen Aman, Shamsullah Burki, Nosheen Noor, Nabiullah

**Affiliations:** 1 Department of Radiology, Lady Reading Hospital-MTI, Peshawar, Pakistan; 2 Department of Occupational Health and Safety, Medical Physics Section, Hamad Medical Corporation, Doha, Qatar; 3 Department of Physics, Qurtuba University of Science and IT, Peshawar, Pakistan

**Keywords:** Acceptable Quality Dose (AQD), Computed tomography (CT), Chest CT, Abdomen CT, Diagnostic reference levels (DRLs)

## Abstract

**Purpose:**

To describe the first experience of patient dose optimization in developing AQD, SSDE and image quality-related DRLs for common CT examinations in the adult age group using the concept of AQD.

**Materials and methods:**

The recent published IQSC from 0 to 4 were used by radiologists for the assessment of image quality. The entire data were collected for five types (brain CT, chest CT, chest HRCT, abdomen KUB CT and abdomen + pelvic CT) CT investigations based on anatomic region (head, chest and abdomen + pelvic). The entire datasets of 264 patients were categorized into three groups based on their weights: group-1 (41–60 kg), group-2 (61–80 kg) and group-3 (81–100 kg). Only score-3 images were considered to assess median and 75th percentile values of CTDI_vol_ and DLP to obtain AQDs and DRLs, respectively.

**Results:**

Following the practical training of four radiologists on image quality scoring criteria for CT images, 264 (92%) out of 288 patient images were clinically acceptable as per IQSC for the study. The AQD (median) values in terms of CTDI_vol_ for the mentioned weight groups were 25.8, 2.7, and 30.6 mGy, while the median DLP values for these groups were 496, 510 and 557 mGycm, respectively, for brain CT. The 75th percentile values in terms of CTDI_vol_ were 30.2, 35.3 and 36.2 mGy, while in terms of DLP, they were 583, 619 and 781 mGycm for brain CT, respectively. Similar results are presented for the above-mentioned procedures, as well as in terms of SSDE.

**Conclusion:**

The first ever experience in obtaining AQD, SSDE and DRLs values for specific CT procedures couples image quality with dose indices, showing comparable values with other relevant studies. Hence, it will provide a baseline for comparison within the facility for future studies and facilitate dose optimization for other facilities aiming for improvement.

## Introduction

The rapid technological advancement in healthcare, particularly the multi-detector computed tomography (MDCT) approach, has made it easier to diagnose with a high degree of accuracy and precision. However, its extensive use is regarded to entail some risk of cancer and other effects from radiation exposure [1, 2]. Healthcare professionals must therefore optimize the acquisition technique to ensure that radiation exposure is as low as reasonably achievable while yet being sufficient to produce the required imaging result.

According to published data, the worldwide demand for computed tomography (CT) imaging performed with multi-detectors has risen sharply in recent years. As per the United Nations Scientific Committee on the Effects of Atomic Radiation (UNCEAR) report 2020/2021, CT makes the largest contribution 61.6% to the overall collective dose [3]. Since there are no dose limits for patients, unlike for occupational radiation workers [4]. Due to increasing number of CT procedures and frequent usage, concerns on radiation dose and its associated health effects are also expanding [5, 6]. Thus, the concept of Diagnostic reference level (DRL) was first introduced by International Commission on Radiological Protection (ICRP) in 1991 [7] and detailed recommendations were given in 1996 [4, 8], which were later introduced by European [9], Ireland [10], USA [11] and other countries.

ICRP defines DRLs as “A form of investigation level, applied to an easily measured quantity, usually the absorbed dose in the air or tissue-equivalent material at the surface of a simple standard phantom or representative patient” [8]. Diagnostic reference levels, in contrast to occupational dosage limits, should not be applied to specific individuals, because the body mass and size of a patient can require a higher dose than those required for a normal patient [4, 12, 13].

Although DRLs are considered as best optimization tool as it cut down exposures higher than the 75th percentile of dose distribution, they should be synchronized with image quality to achieve the desired goal of ALARA (As Low as Reasonably Achievable). Otherwise, several shortcomings may arise, such as being considered a “speed limit”, the misconception that being below the DRLs indicates optimization is achieved, neglecting image quality during DRLs estimation and occasionally being perceived as a dose limit, as reported in several studies [4, 13–15].

To address these limitations of DRLs, a new concept called acceptable quality dose (AQD) was introduced [13], which correlates image quality with dose indices. This requires first assessing the image quality by the radiologist and then analyzing dose indices only for images that are considered acceptable [4]. Thus, prioritizing image quality becomes the primary goal rather than patient radiation dose. Furthermore, the image quality scoring criteria (IQSC) for pediatric CT have been provided [4, 16] and this concept was applied to adult imaging in this study.

Hence, using the novel concept of AQD and IQSC, this study was conducted for the first time at the Department of Radiology, Lady Reading Hospital-Medical Teaching Institute (LRH-MTI), Peshawar, Khyber Pakhtunkhwa (KP). Thus, the main purpose of this study was to develop a baseline for AQD, Size Specific Dose Estimate (SSDE) and image quality-related DRLs for common CT procedures, to address the shortcomings of DRLs and to avoid unnecessary repetition of CT examinations, ensuring a safe culture toward dose optimization in accordance with the ALARA principle.

## Materials and methods

This retrospective study was conducted in the Radiology Department at Lady Reading Hospital (LRH), Peshawar Pakistan during the period from March to June, 2022. A total of 264 patient data with a score of 3 were acquired from individuals who underwent various CT procedures, including brain, chest, chest HRCT, abdomen KUB, and abdomen + pelvic classified into three anatomic regions (brain, chest and abdomen + pelvis) performed using Canon 160 Slice Aquiline Prime Series CT scanner. Patients having age less than 18 years were excluded from the study. The demographic data of patients i.e., age, sex, weight, height and dosimetric data CTDI and DLP, along with image quality score were recorded in a dedicated performa.

To determine size-specific AQD and image quality-related DRL, the entire dataset of 264 patients was categorized into three groups based on their weights: group-1 (41–60 kg), group-2 (61–80 kg) and group-3 (81–100 kg), as recommended by Rehani [14]. The radiation doses received by patients during CT scans depend significantly on the output of scanner and patient’s size. Therefore, the concept of size-specific dose estimate (SSDE) was introduced by American Association of Physicists in Medicine (AAPM) report 204 [17], mainly aiming to estimate patient doses for each scanning region, taking into account patient’s size.

For SSDE estimation, the effective diameter of the corresponding body region is calculated from anteroposterior (AP) and lateral thickness of large slice of body region image using Picture Archiving Communication System (PACS). The value of SSDE for the body region is estimated using the conversion factor corresponding to the effective diameter from AAPM report 204, while the SSDE for head region is estimated using corresponding conversion factor for various head circumference reported by Tiao Chen et al*.,* respectively [18].

Similarly, effective doses calculated for group-2 having standard body weight (70 ± 10 kg) [19], are measure of the risk associated with heterogeneous radiation exposure, expressed in terms of equivalent whole-body exposure multiplied by a factor called k factor, following ICRP report 102 [20]. Empirical effective doses are calculated using the formula mentioned below;

$$\text{Effective} \text{dose} \,(\text{mSv})=k \times \text{DLP} \,(\text{mGy}. \text{cm})$$

where k is a body region-based empirical weighting factor (mSv mGy^-1^ cm^-1^).

The conversion factor 0.0012, 0.014 and 0.015 mSv mGy^−1^ cm^−1^ were used for brain, chest and abdomen + pelvic, respectively.

The Image Quality Scoring Criteria [4, 16] provided for pediatrics were used in this study for establishing AQD and DRLs for adults. Radiologists were briefed about Image Quality Scoring Criteria for subjective assessment of image quality. Briefly, the scoring criteria and scale ranging from 0 to 4 were established based on a recent study [4] and used by radiologists for the purpose of assessing and scoring image quality as below:

The score of 0 s was assigned when the region of interest was not included in the image, a score of 1 when the desired diagnostic interpretations couldn’t be ruled out due to unacceptable image quality; a score of 2 was made when limited clinical interpretations were possible due to noisy images, while score-3 was assigned to images with adequate quality and final score of 4 was given to high-quality images with minimal noise.

**Fig. 1:**
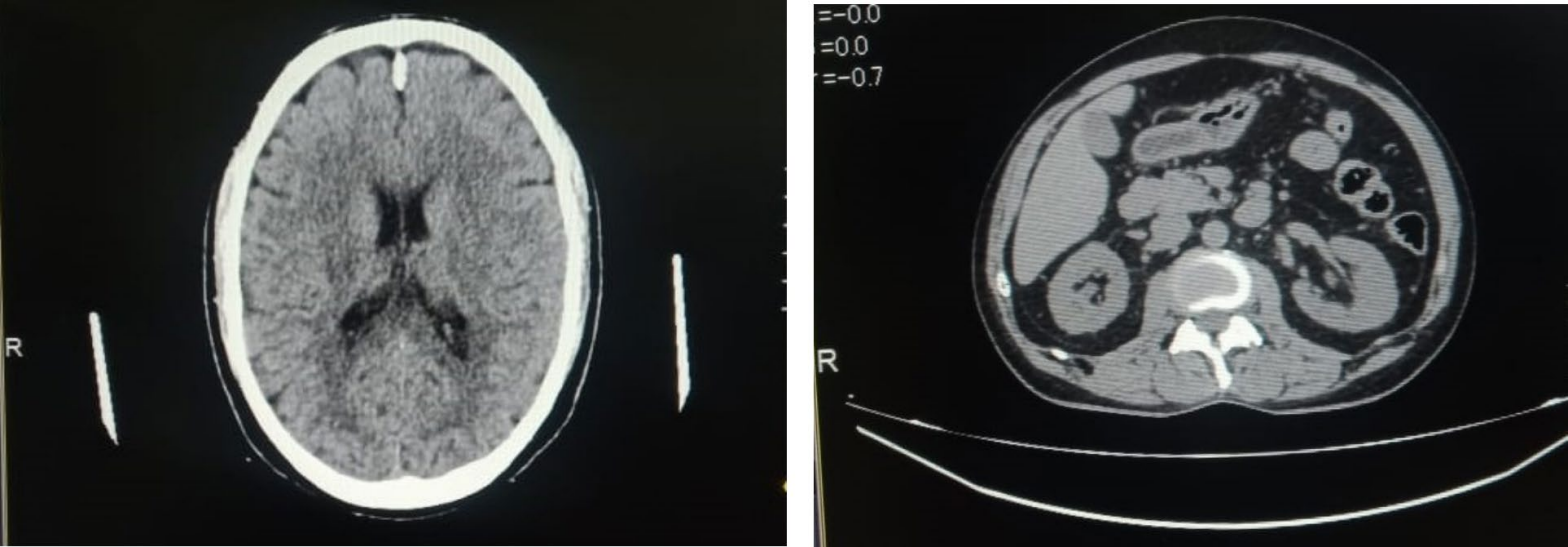
Left and right images representing score of 3 and 4, respectively

The following two images taken during the study from Canon 160 Slice Aquiline Prime Series CT scanner representing the scores of 3 and 4, as shown in Fig. 1, respectively. The first (left) image exhibits more noise but contains significant diagnostic information, while the second (right) image is of good quality with negligible noise.

### Inter-observer agreement

To examine the inter-observer agreement among the participating radiologists having more than 10 years of experience and to assess their experience of image quality scoring as per the set criteria, 60 randomly selected images i.e., 20 images from each of the three types of investigations head, chest and abdomen + pelvic respectively, were reviewed. All radiologists were provided access to the better and identical environment, which consisted of PACS monitor that enabled them to manipulate the window settings of the images for optimal viewing according to their preferences.

After finding substantial agreement between the participating radiologists, data were collected for those CT images that had an acceptable image quality score of 3.

## Results

**Fig. 2:**
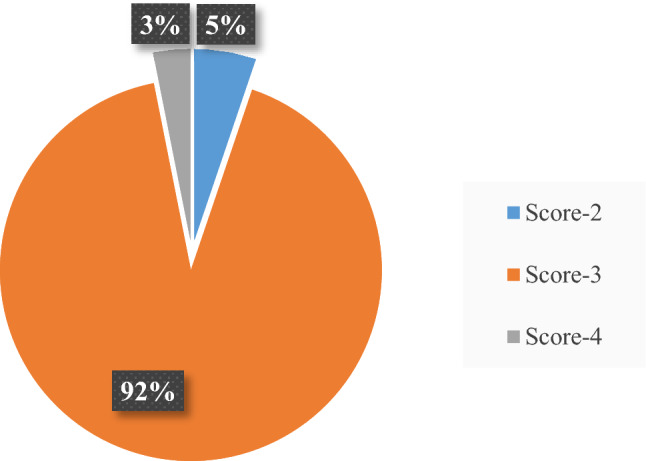
Distribution of data in terms of score-2, 3 and 4

Data were collected in accordance with the idea of AQD. Only those CT images with an acceptable image quality score of 3 were included for data analysis. There were a total of 288 patients and out of those, 24 images (about 8%) had a quality score that was different from 3 (15 patients with score of 2 and 9 patients with score-4, no patients score less than 2) as shown in Fig. 2. Therefore, they were excluded from AQD estimation.

**Fig. 3:**
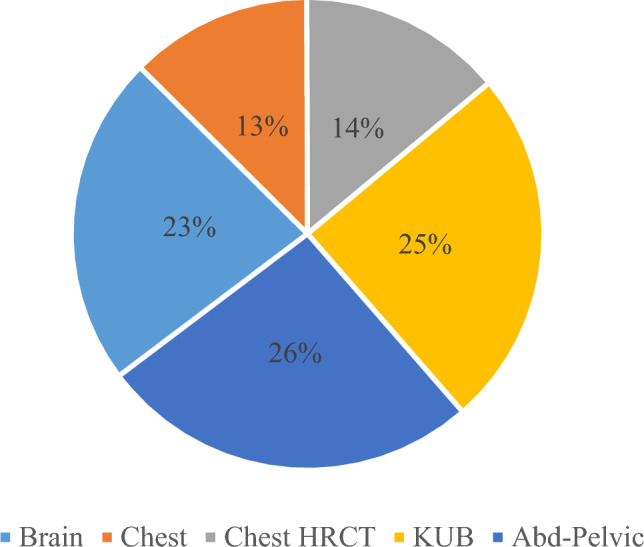
Distribution of procedures in percentage as per anatomic part

The CT scans of the 264 patients, which had an acceptable degree of image quality (score-3) were divided into brain, chest, chest HRCT, abdomen KUB, and abdomen-pelvic CTs, with respective numbers of 60, 33, 37, 65, and 69. Among the performed procedures per type, abdomen + pelvic had the highest 26.1%, followed by abdomen KUB 24.6%, brain 22.7%, chest HRCT 14% and chest 12.5%, as shown in Fig. 3.

**Fig. 4:**
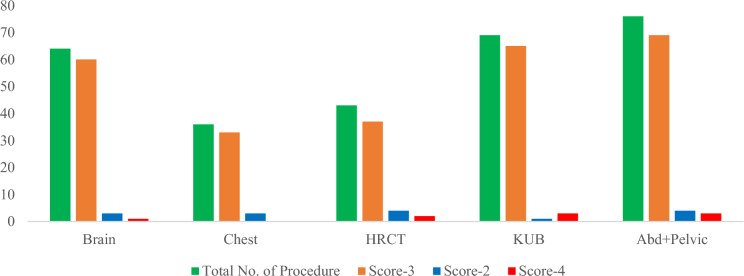
Breakdown of each procedure in terms of total number of procedures and scores-2, 3 and 4

The scoring criteria (2, 3 and 4) for each procedure were further divided as follows: for brain, representing 5%, 94% and 1% respectively, and for the chest, they were 8%, 92% and 0%. Similar results were observed for chest HRCT (9%, 86%, and 5%), abdomen KUB (1%, 94% and 5%) and abdomen + pelvic (5%, 91% and 4%), respectively. The overall scores of each procedure in terms of scoring criteria (2, 3 and 4) are provided in Fig. 4.

**Fig. 5:**
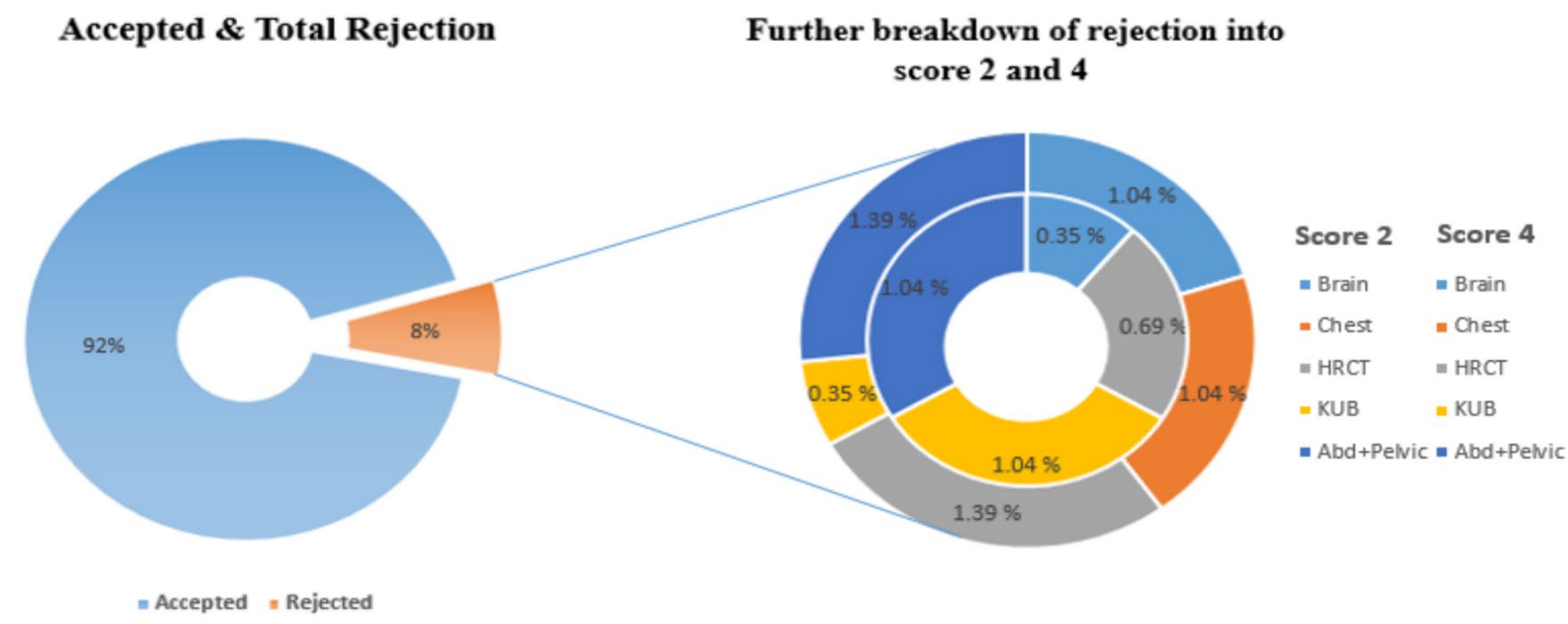
Illustration of accepted and total rejection with further segregation into score-2 (outside) and score-4 (inside) represented in percentage, respectively

The criteria for evaluating image quality introduced a novel concept of Repeat Reject Analysis (RRA) within the realm of CT. In diagnostic imaging, one of the main goals of a quality assurance (QA) program is to produce consistent high-quality images at a minimum exposure to the patient. It reduces the cost, workload, and radiation exposure to patients and personnel [21]. The comprehensive breakdown of total rejections along with further details is presented in Fig. 5.

The inter-observer agreement between the reporting radiologists showed substantial agreement (kappa value 0.77–0.89) through kappa statistics as the median image quality scores for all the CT exams (head, chest and abdomen + pelvic) was 3 for all 60 patients as shown in Table 1.

**Table 1:** Median of mage quality score for three common CT procedures

Procedure name	Group-1	Group-2	Group-3	Group-4	Median
Head (20)	3	3	3	3	3
Chest (20)	3	3	3	3	3
Abdomen + pelvis (20)	3	3	3	3	3

Similarly, the score of 3 was provided by all four radiologists for 80% of all CT exams, while 2.91% of CT exams received a score of 4 and 17.08% of exams received a score of 2 as summarized in Table 2.

**Table 2:** Frequency of subjective image quality score (1–4) for the four groups of radiologists

Procedure name	Score	Group-1 (%)	Group-2 (%)	Group-3 (%)	Group-4 (%)
Head (20)	1	0 (0)	0 (0)	0 (0)	0 (0)
	2	2 (10)	1 (5)	1 (5)	1 (5)
	3	17 (85)	19 (95)	17 (85)	19 (95)
	4	1 (5)	0 (0)	2 (10)	0 (0)
Chest (20)	1	0 (0)	0 (0)	0 (0)	0 (0)
	2	6 (30)	8 (40)	7 (35)	6 (30)
	3	14 (70)	12 (60)	13 (65)	13 (65)
	4	0 (0)	0 (0)	0 (0)	1 (5)
	1	0 (0)	0 (0)	0 (0)	0 (0)
Abdomen + pelvic (20)	2	2 (10)	2 (10)	2 (10)	3 (15)
	3	17 (85)	17 (85)	18 (90)	16 (80)
	4	1 (5)	1 (5)	0 (0)	1 (5)

SPSS version 20 was used to analyze all data of score-3 for brain, chest, chest HRCT, abdomen KUB and abdomen-pelvic. The mean, AQD (median), the 75th percentile (facility DRLs) in terms of CTDI_vol_ and DLP were determined. Similarly, SSDE and effective doses (for group-2 only) were calculated, as shown in Tables 3, 4, 5, 6 and 7, respectively, for each procedure.

**Table 3:** Analysis of patient distribution in different weight groups for brain study, including SSDE, 75th percentile, CTDI and DLP per weight group (AQD)

Procedure—brain CT				
Weight groups		41–60 kg	61–80 kg	81–100 kg
No. of patients *n* = 60		26 (43.4%)	24 (40%)	10 (16.6%)
Ave. age (year)		29	44	52
Ave. HC* (cm)		48	52	51
CTDI_vol_ mGy	Median	25.8	25.7	30.6
	75th percentile (DRL)	30.2	35.3	36.2
	Mean ± SD	26.4 ± 6	27.4 ± 11	30.8 ± 9
DLP mGycm	Median	496	510	557
	75th percentile (DRL)	583	619	781
	Mean ± SD	496 ± 141	561 ± 316	603 ± 219
SSDE mGy	Median	23.7	22.3	27.6
	75th percentile (DRL)	29.3	31.3	31.9
	Mean ± SD	24.8 ± 6	23.9 ± 10	27.1 ± 8
Effective dose mSv	Ave. Effective dose	–	1.2	–

^*^HC Head circumference

**Table 4:** Analysis of patient distribution in different weight groups for chest study, including SSDE, 75th percentile, CTDI and DLP per weight group (AQD)

Procedure—chest CT				
Weight groups		41–60 kg	61–80 kg	81–100 kg
No. of patients *n* = 33		11 (33.3%)	12 (36.4%)	10 (30.3%)
Ave. Age (Year)		32	48	54
Ave. ED* (cm)		23	28	28
CTDI_vol_ mGy	Median	8.5	8.7	14.3
	75th percentile (DRL)	11.4	14.6	17.9
	Mean ± SD	9 ± 3	10.3 ± 5	14.9 ± 3
DLP mGy cm	Median	388	377	605
	75th percentile (DRL)	501	555	822
	Mean ± SD	381 ± 151	448 ± 311	691 ± 262
SSDE mGy	Median	12.2	13.9	19.5
	75th percentile (DRL)	18.2	20	23.7
	Mean ± SD	13.7 ± 5	15.4 ± 7	19.7 ± 5
Effective dose mSv	Ave. effective dose	–	6.3	–

^*^ED Effective diameter

**Table 5:** Analysis of patient distribution in different weight groups for chest HRCT study, including SSDE, 75th percentile, CTDI and DLP per weight group (AQD)

Procedure—chest HRCT				
Weight groups		41–60 kg	61–80 kg	81–100 kg
No. of patients *n* = 37		16 (43.2%)	11 (29.8%)	10 (27%)
Ave. age (year)		39	47	63
Ave. ED* (cm)		23	27	28
CTDI_vol_ mGy	Median	7.5	10.7	13.7
	75th percentile (DRL)	12.7	15.9	18.9
	Mean ± SD	9.1 ± 6	11.3 ± 7	14.8 ± 6
DLP mGycm	Median	220	448	538
	75th percentile (DRL)	427	665	744
	Mean ± SD	307 ± 200	430 ± 262	568 ± 219
SSDE mGy	Median	12.1	13.2	18.1
	75th percentile (DRL)	17.2	19.6	24.9
	Mean ± SD	13.4 ± 9	15.8 ± 11	19.4 ± 6
Effective Dose mSv	Ave. Effective dose	–	6	–

^*^ED Effective diameter

**Table 6:** Analysis of patient distribution in different weight groups for abdomen KUB study, including SSDE, 75th percentile, CTDI and DLP per weight group (AQD)

Procedure—Abdomen KUB CT				
	Weight groups	41–60 kg	61–80 kg	81–100 kg
No. of patients *n* = 65		34 (52.3%)	19 (29.2%)	12 (18.5%)
Ave. age (year)		28	43	54
Ave. ED* (cm)		23	28	29
CTDI_vol_ mGy	Median	6.5	6.8	13.9
	75th percentile (DRL)	8.6	11	19.4
	Mean ± SD	7.8 ± 5	9.5 ± 6	13.9 ± 6
DLP mGycm	Median	323	350	624
	75th percentile (DRL)	444	539	936
	Mean ± SD	378 ± 242	481 ± 281	678 ± 273
SSDE mGy	Median	9.8	9.5	16.4
	75th percentile (DRL)	13.3	16.3	24.8
	Mean ± SD	12 ± 8	12.7 ± 8	17.5 ± 7
Effective dose mSv	Ave. effective dose	–	7.2	–

*ED Effective diameter

**Table 7:** Analysis of patient distribution in different weight groups for abdomen + pelvic study, including SSDE, 75th percentile, CTDI and DLP per weight group (AQD)

Procedure—abdomen-pelvic CT				
Weight groups		41–60 kg	61–80 kg	81–100 kg
No. of patients *n* = 69		30 (43.5%)	23 (33.3%)	16 (23.2%)
Ave. age (year)		31	48	56
Ave. ED* (cm)		23	28	28
CTDI_vol_ mGy	Median	8.2	11.6	18.4
	75th percentile (DRL)	16.2	18.2	19.8
	Mean ± SD	10.7 ± 6	12.4 ± 5	17.4 ± 4
DLP mGycm	Median	449	679	798
	75th percentile (DRL)	801	1064	1169
	Mean ± SD	522 ± 301	680 ± 324	891 ± 270
SSDE mGy	Median	12.8	13.8	25.2
	75th percentile (DRL)	23.1	23.3	27.1
	Mean ± SD	16.4 ± 8	16.6 ± 8	23 ± 6
Effective dose mSv	Ave. effective dose	–	10.2	–

^*^ED Effective diameter

## Discussion

DRLs based on image quality assessment are the best optimization tool that plays a key role in optimization of patient doses and are also challenging task for technologists in patient dose reduction keeping image quality acceptable for diagnosis. Therefore, this study was conducted for the first time to establish AQD, image quality-related DRLs and SSDE for commonly performed CT procedures for adults of above 18 years as facility DRLs were not established earlier.

The concept of AQD and IQSC was employed by radiologists to assess the image quality and only images of acceptable quality were then used for dose analysis. It addressed the short comings of DRLs that were pointed out by Rehani [13] and the concept applied for the first time in Qatar [4] for pediatric study.

Certain interesting findings were revealed in this study. Initially, using the concept of AQD and IQSC, 264 out of 288 (almost 92%) CT exams were clinically acceptable to radiologists and had score of 03 while 24 (almost 8%) were excluded form dose analysis as they had score of 2 (5%) and 4 (3%), as shown in Fig. 2. Specifically, score-2 (nearly 5%) is not trivial as they were repeated. This suggests that there is a probability that a considerable number of exams that are not clinically acceptable are also counted in most dose surveys where image quality is not assessed and documented and the dose indices reported will not provide real values of clinically acceptable exams [4].

Image quality assessment sparked a new concept of Repeat Rejection Analysis (RRA) in CT that was not previously reported. Since, CT exposes patients to higher doses, has longer cycle times, and requires larger capital investment than either radiography or mammography, yet RRA for CT is neither suggested nor mandated by any regulatory bodies or professional organizations. In the current study repeat rate was observed 5% in the form of score-2 for CT procedures and 3% was of high-quality images (score-4) though it was not repeated but contributed unnecessary higher radiation dose to the patient. Rejection rate of the current study was 5% which is lower than AAPM [22] recommending limit of 10% for radiography while higher reported by Sean rose et al. 1.2% for CT [23]. Further breakdown of rejection in specific procedures is illustrated in Fig. 5.

Determination of rejection rate is crucial since it reveals the underlying causes for rejection which can be addressed in a better way to improve the image quality, lower the rejection rate, decrease patient radiation exposure, cut down cost, optimize machine performance, life along with reduction of staff burden and long waiting list.

For Brain CT, the median CTDI_vol_ values for group-1, 2 and 3 were 25.8, 25.7 and 30.6 mGy while median DLP values were 496, 510 and 557 mGycm, respectively. The 75th percentile CTDI_vol_ values for these groups were 30.2. 35.3 and 36.2 mGy and DLP values were 583, 619 and 781 mGycm, respectively. Similarly, the median SSDE values were calculated as 23.7, 22.3 and 28.8 mGy, while 75th percentile values were 29.3, 31.3 and 31.9 mGy, respectively. The effective dose calculated for group-2 was 1.2 mSv. All these values, mean, standard deviation and average circumference of head have been presented in Table 3.

**Fig. 6:**
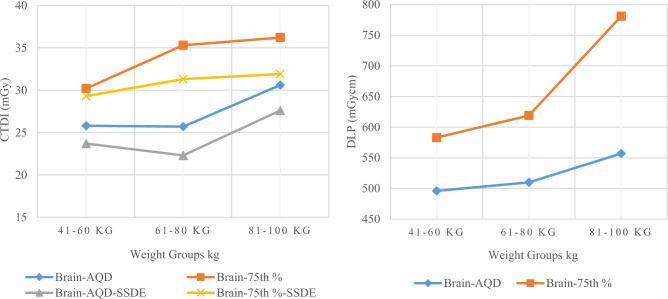
The image on the left displays AQD, 75th % of brain CT in terms of CTDI and SSDE, while the image on the right displays AQD and 75th % in terms of DLP, respectively

As it clearly observed from Fig. 6, when the weight increases from goup-1 to group-3, exposure parameters (CTDI_vol_ and DLP) increase proportionally. The size of the head remains relatively stable, showing only minor changes despite increases in age and weight. Therefore, a slight increase was observed in SSDE, CTDI_vol_ and DLP values among the weight groups verifying our findings. All values approximately consistent between group-1 and 2 in terms of CTDI_vol_ while a significant rise was observed in terms of both CTDI_vol_ and DLP in group-3 due to increased head dimensions and range compared to group-1 and 2.

**Fig. 7:**
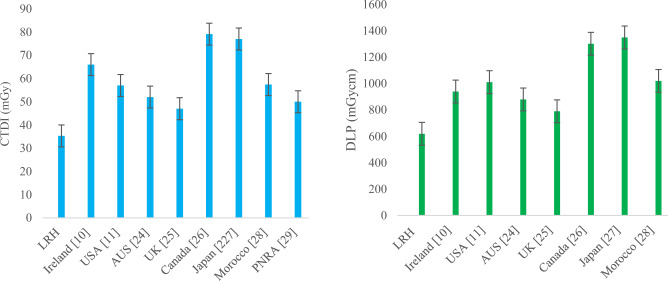
Comparison of DRLs in terms of CTDI (left) and DLP (Right) of LRH (group-2) brain CT with other relevant studies

The median and 75th percentile values of CTDI_vol_ and DLP become brain AQDs and DRLs for their corresponding weight groups, respectively. Similarly, the median and 75th percentile values in terms of SSDE become brain AQDs of SSDE and DRLs of SSDE for their corresponding weight groups, respectively. By comparing facility DRL (75th percentile value) of group-2 brain CT in terms of CTDI_vol_ and DLP with other countries DRL such as Ireland [10], USA [11], Australia [20], UK [21], Canada [22], Japan [23] and Morocco [24], our facility DRL was found to be very low, as shown in Fig. 7, respectively. Facility DRL was also low from national DRLs suggested by regulatory body PNRA in the country [25].

This reveals that prioritizing image quality over dose indices results in considerably lower doses without compromising image quality, showcasing safe practices and dose optimizations protocols being followed during imaging procedures.

For chest CT, the median CTDI_vol_ values for group-1, 2 and 3 were 8.5, 8.7 and 14.3 mGy while median DLP values were 388, 377 and 605 mGycm, respectively. The 75th percentile CTDI_vol_ values for these groups were 11.4, 14.6 and 17.9 mGy and DLP values were 501, 555 and 822 mGycm, respectively. Similarly, the median SSDE values were calculated as 12.2, 13.9 and 19.5 mGy, while 75th percentile values were 18.2, 20 and 23.7 mGy, respectively. The effective dose calculated for group-2 was 6.3 mSv. All these values, mean, standard deviation of CTDI_vol_ and DLP have been presented in Table 4.

**Fig. 8:**
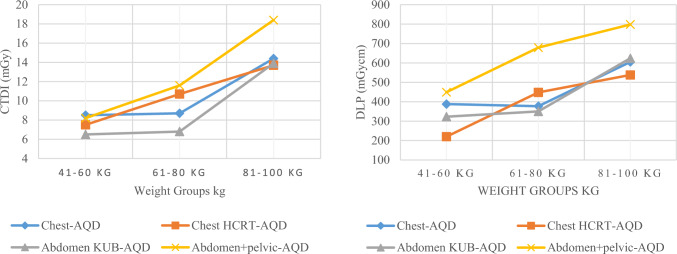
The images display AQD (median) in terms of CTDI_vol_ (left) and DLP (right) for chest, chest HRCT, abdomen KUB, and abdomen + pelvic CTs, respectively

**Fig. 9:**
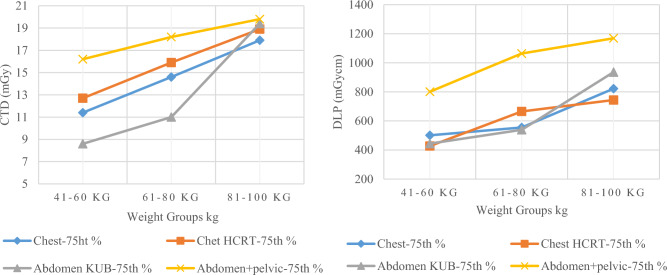
The images display 75th % in terms of CTDI_vol_ (left) and DLP (right) for chest, chest HRCT, abdomen KUB, and abdomen + pelvic CTs, respectively

**Fig. 10:**
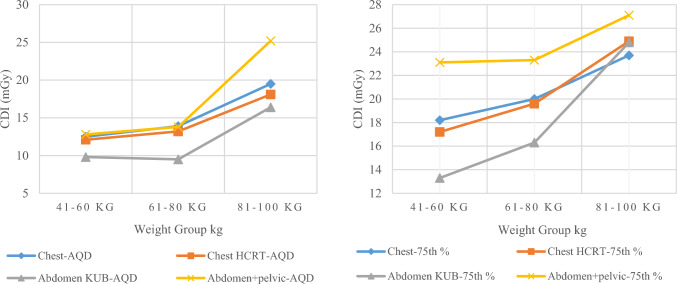
The images display AQD-median (left) and 75th % SSDE (right) for chest, chest HRCT, abdomen KUB and abdomen + pelvis in terms of SSDE, respectively

A gradual increase was noticed in median and 75th percentile values in terms of CTDI_vol_ and DLP, especially in group-3, as patient’s weight was higher compared to group-1 and 2, as shown in Figs. 8 and 9. A similar result was noticed for SSDE value, which can be seen in Fig. 10. When weight increases, a correspondingly increase in exposure parameter is required to achieve adequate quality for accurate diagnosis, as evident from Fig. 8, 9 and 10. That is why the curve in Figs. 8, 9 and 10 gets steeper as weight increases.

**Fig. 11:**
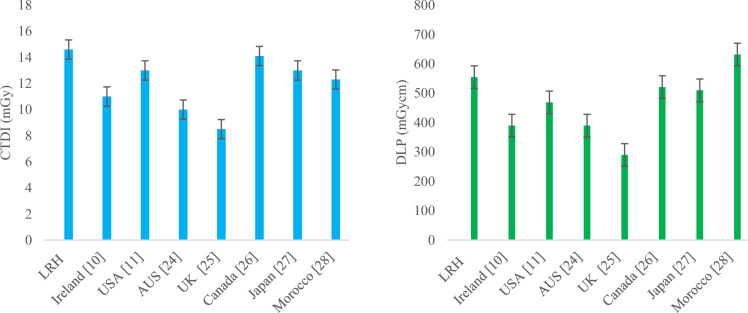
Comparison of DRLs in terms of CTDI (left) and DLP (Right) of LRH (group-2) chest CT with other relevant studies

The comparison of group-2 chest CT presented in Fig. 11 shows that facility DRLs in terms of CTDI were comparable to those of the USA, Canada, and Morocco’s DRL, whereas they were higher than DRLs of UK, Australia, Ireland and Morocco. In terms of DLP, DRLs of LRH chest CT were comparable to those of Canada and Japan’s DRL and less than Morocco’s, while higher than those of Ireland, the USA, Australia and the UK’s.

The possible reasons behind the higher DRL for chest CT are the demand for high-quality images from reporting radiologists. It was challenging to report low-quality images since it was our first experience of optimizing patient doses in terms of quality acceptable for radiologist, rather than merely meeting dose requirements. This often result in repetition of procedure. Thus, it emphasizes the need for rigorous training of radiologists on image quality assessments, prioritizing the acceptance of images with some noise over unnecessarily crisp images.

For chest HRCT, the median CTDI_vol_ values for group-1, 2 and 3 were 7.5, 10.7 and 13.7 mGy while median DLP values were 220, 448 and 538 mGycm, respectively. The 75th percentile CTDI_vol_ values for these groups were 12.7, 15.9 and 18.9 mGy and DLP values were 427, 665 and 744 mGycm, respectively. Similarly, the median SSDE values were calculated as 12.1, 13.2 and 18.1 mGy, while 75th percentile values were 17.2, 19.6 and 24.9 mGy, respectively. The effective dose calculated for group-2 was 6 mSv. All these values, mean, standard deviation of CTDI_vol_ and DLP have been presented in Table 5.

**Fig. 12:**
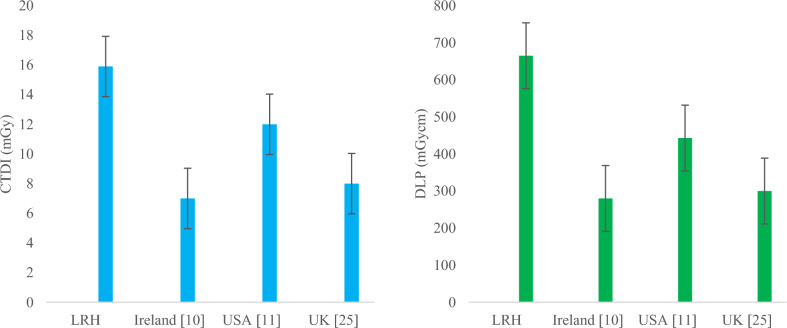
Comparison of DRLs in terms of CTDI (left) and DLP (Right) of LRH (group-2) chest HRCT with other relevant studies

A proportional increase in the median and 75th percentile values of CTDI_vol_, DLP and SSDE for chest HRCT observed, as evident from Figs. 8, 9 and 10, respectively, as weight gets higher from group-1 to group-3. Comparing The 75th percentile values of chest HRCT for group-2 with other studies, our results show higher values than the DRLs of Ireland, the USA and the UK, both in terms of CTDI_vol_ and DLP, as illustrated in Fig. 12. This study was conducted in a public sector hospital with high influx of patient undergoing CT procedures. It emphasizes the need for proper training of CT technologists in dose optimization protocols and encourages radiologists to report on images of adequate quality. Moreover, further optimization is needed to ensure patient safety.

For abdomen KUB CT, the median CTDI_vol_ values for group-1, 2 and 3 were 6.5, 6.8 and 13.9 mGy while median DLP values were 323, 350 and 624 mGycm, respectively. The 75th percentile CTDI_vol_ values for these groups were 8.6, 11 and 19.4 mGy and DLP values were 444, 539 and 936 mGycm, respectively. Similarly, the median SSDE values were calculated as 9.8, 9.5 and 16.4 mGy, while 75th percentile values were 13.3, 16.3 and 24.8 mGy, respectively. The effective dose calculated for group-2 was 7.2 mSv. All these values, mean, standard deviation of CTDI_vol_ and DLP have been presented in Table 6.

Similarly, for abdomen + pelvic CT, the median CTDI_vol_ values for group-1, 2 and 3 were 8.2, 11.6 and 18.4 mGy while median DLP values were 449, 679 and 798 mGycm, respectively. The 75th percentile CTDI_vol_ values for these groups were 16.2, 18.2 and 19.8 mGy and DLP values were 801, 1064 and 1169 mGycm, respectively. Similarly, the median SSDE values were calculated as 12.8, 13.8 and 25.2 mGy, while 75th percentile values were 23.1, 23.3 and 27.1 mGy, respectively. The effective dose calculated for group-2 was 10.2 mSv. All these values, mean, standard deviation of CTDI_vol_ and DLP have been presented in Table 7.

**Fig. 13:**
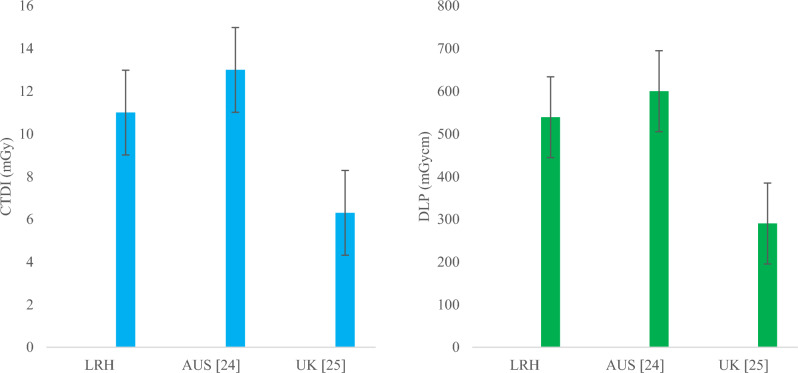
Comparison of DRLs in terms of CTDI (left) and DLP (Right) of LRH (group-2) abdomen KUB CT with other relevant studies

A gradual increase was also observed in both abdomen KUB and abdomen + pelvic CT studies, as shown in Figs. 8, 9 and 10, respectively. Comparing the 75th percentile values with those of other relevant studies, the DRL of abdomen KUB for group-2 exhibited lower values in terms of CTDI_vol_ and DLP compared to Australia, while it was higher than that of the UK, as illustrated in Fig. 13. However, this can be further restricted, as abdomen KUB is primarily performed for Renal stone localization, which can be visualized at low exposure and differentiated from other pathologies and lesions even in low-quality images [30].

**Fig. 14:**
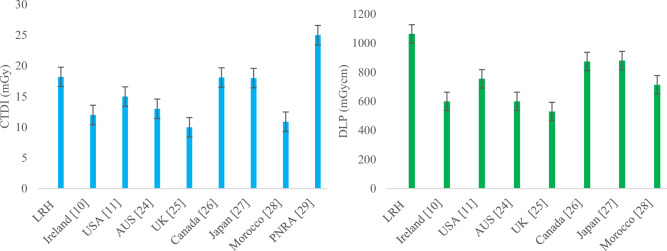
Comparison of DRLs in terms of CTDI (left) and DLP (Right) of LRH (group-2) abdomen + pelvis CT with other relevant studies

Similarly, 75th percentile value of abdomen + pelvic of group-2 in terms of CTDI_vol_ was comparable to Canada and Japan but lower than national DRL of PNRA. Regarding DLP, 75th percentile value of abdomen + pelvic was relatively higher than that of all relevant studies, as shown in Fig. 14. The raised values in exposure parameters (CTDI_vol_ and DLP) are due to the requirement of high resolution and quality images demanded by radiologists. This requirement aims to missing minor pathologies and to prevent the unnecessary repetition of procedures. In addition, the unavailability of relevant facility DRL (FDRL) from the institute, which could have provided guidance during CT procedures, contributes to these high values.

**Fig. 15:**
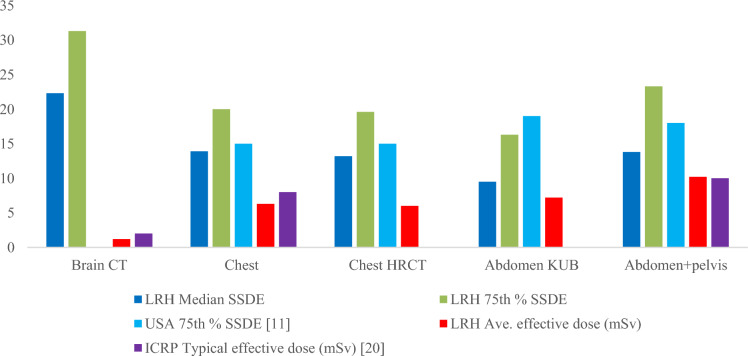
Comparison of 75th % SSDE and effective dose of group-2 with USA and ICRP for Brain, chest, chest HRCT, abdomen KUB and abdomen + pelvic, respectively

For SSDE, the 75th percentile SSDE values of group-2 were compared with those of the USA, as depicted in Fig. 15. The 75th percentile values for chest and chest HRCT were slightly higher than those in the USA. Conversely, for abdomen KUB, the 75th percentile value was lower than that of the USA, whereas for abdomen + pelvic, it was higher. All these values were compared with the USA SSDE values, which had a diameter range from 29-33 cm, comparable to the effective diameter range of group-2 for corresponding procedures. Specifically, group-2, chest had an average effective diameter of 28 cm, chest HRCT 27 cm, abdomen KUB and abdomen-pelvic had an effective diameter of 28 cm as can be seen in Table 4–7, respectively.

Similarly, average effective doses of group-2 were compared with ICRP typical effective doses, showing that the effective doses of the Brain and chest were lower, while abdomen + pelvic value was approximately equal. Since, our study includes pelvic with the abdomen; this results in a slightly higher dose than abdomen KUB, as the combined and pelvic scan covered a wide range.

All the median and 75th percentile values of CTDI_vol_, DLP become facility AQDs and DRLs for their corresponding weight groups, respectively. Similarly, the median and 75th percentile values in terms of SSDE become AQDs of SSDE and DRLs of SSDE for their corresponding weight groups, respectively.

However, with the current values established, further work can be conducted to lower them once the staff are fully adapted to applying dose optimization protocols in daily routine procedures and the radiologists adhered to reporting and diagnosis at adequate image quality rather than demanding high-quality images. Thus, the AQD concept relies on the training and orientation of both technologists and radiologists. This is the beauty of AQD: it brings attention to the quality of the image, which directly affects patient dose and discourages the pursuit of unnecessary high quality. This represents a significant step toward fostering a culture of safe radiation practices.

## Conclusion

The concept of AQD and DRLs based on image quality has numerous benefits as quality is prioritized over dose. This study was distinctive in a way that ADQ and DRLs are developed at the image quality acceptable to radiologists for diagnosis which is the true presentation of dose optimization and alerts facility about their rejection rate. In addition, it also addresses the technical gap between CT technologists and radiologists that causes them to focus on image quality levels that further develop their skills regarding dose optimization.
